# Fully integrated reflection-mode photoacoustic, two-photon, and second harmonic generation microscopy *in vivo*

**DOI:** 10.1038/srep32240

**Published:** 2016-08-31

**Authors:** Wei Song, Qiang Xu, Yang Zhang, Yang Zhan, Wei Zheng, Liang Song

**Affiliations:** 1 Research Laboratory for Biomedical Optics and Molecular Imaging, Shenzhen Key Laboratory for Molecular Imaging, Institute of Biomedical and Health Engineering, Shenzhen Institutes of Advanced Technology, Chinese Academy of Sciences, Shenzhen 518055, China; 2 Brain Cognition and Brain Disease Institute, Shenzhen Institutes of Advanced Technology, Chinese Academy of Sciences, Shenzhen 518055, China

## Abstract

The ability to obtain comprehensive structural and functional information from intact biological tissue *in vivo* is highly desirable for many important biomedical applications, including cancer and brain studies. Here, we developed a fully integrated multimodal microscopy that can provide photoacoustic (optical absorption), two-photon (fluorescence), and second harmonic generation (SHG) information from tissue *in vivo*, with intrinsically co-registered images. Moreover, using a delicately designed optical-acoustic coupling configuration, a high-frequency miniature ultrasonic transducer was integrated into a water-immersion optical objective, thus allowing all three imaging modalities to provide a high lateral resolution of ~290 nm with reflection-mode imaging capability, which is essential for studying intricate anatomy, such as that of the brain. Taking advantage of the complementary and comprehensive contrasts of the system, we demonstrated high-resolution imaging of various tissues in living mice, including microvasculature (by photoacoustics), epidermis cells, cortical neurons (by two-photon fluorescence), and extracellular collagen fibers (by SHG). The intrinsic image co-registration of the three modalities conveniently provided improved visualization and understanding of the tissue microarchitecture. The reported results suggest that, by revealing complementary tissue microstructures *in vivo*, this multimodal microscopy can potentially facilitate a broad range of biomedical studies, such as imaging of the tumor microenvironment and neurovascular coupling.

Over the past few decades, optical microscopy has become an indispensable technology in biophysical research. It offers great advantages in investigating physiological and pathological processes by revealing the morphological, functional and molecular features of biological specimens with cellular and subcellular spatial resolution. Based on various imaging contrasts within tissues, including optical scattering, fluorescence, and polarization, several optical microscopic technologies have been established and broadly applied in neurology^1^,^2^, oncology^3^,^4^, vascular biology^5^,^6^, and ophthalmology^7^,^8^.

Among all these optical microscopic technologies, multiphoton fluorescence microscopy and second harmonic generation (SHG) microscopy are capable of providing specific types of molecular imaging information at diffraction-limited spatial resolution in deep positions of solid tissues, significantly facilitating research in biological sciences and medicine^3^,^4^,^5^,^9^. By detecting the fluorescence emission from intrinsic chromophores, such as cellular reduced nicotinamide adenine dinucleotide (NADH) and tryptophan, multiphoton microscopy can reveal cellular/subcellular morphology, cellular metabolism, and protein characteristics within different tissue microenvironments, greatly benefiting premalignancy diagnosis^10^,^11^. Additionally, many genetic manipulation approaches that involve the engineering of optical reporter genes or proteins into the promoter region of a gene of interest have already been developed, and a variety of transgenic mouse models have been created that express fluorescent proteins (for example, green fluorescent protein, GFP) within specific cells and/or organs^12^. As a result, multiphoton microscopy enables longitudinal monitoring of physiological processes in small animals *in vivo*. SHG microscopy is able to distinguish the microanatomical signatures of tissue under normal and malignant conditions based on the noncentrosymmetric properties of endogenously orientated biological structures, such as collagen and microtubules^13^,^14^. For example, the textural features of the organized collagen matrix underlying the epithelia can be delineated in three-dimensional space; therefore, SHG microscopy enables accurate evaluation of early-stage squamous epithelial cancers. However, the main limitation of these two imaging modalities is that they fail to directly measure the optical absorption; this information is strongly associated with the physiological and pathological status of tissues.

In contrast, photoacoustic (PA) imaging, which acoustically detects pressure waves induced by the transient thermo-elastic expansion due to the absorption of pulsed optical energy by biomolecules, is very sensitive in mapping the optical absorption properties of biological tissue^15^,^16^. Consequently, the molecular specificity of nonfluorescent intrinsic chromophores, such as hemoglobin, melanin, and lipids, provides information complementary to that of the well-established imaging technologies based on fluorescence and/or SHG imaging mechanisms. Optical-resolution photoacoustic microscopy (OR-PAM), an important branch of photoacoustic imaging technology, uses visualization at the optical diffraction-limited lateral resolution down to the micrometer or even submicrometer scale by utilizing a microscope objective to tightly focus the photoacoustic excitation laser onto biological samples^17^,^18^,^19^. OR-PAM is capable of mapping, without labels, the volumetric distribution of optical absorption with fine spatial resolution. Furthermore, adopting the well-established spectroscopic photoacoustic measurement of chromophore concentration, PAM quantifies many critical physiology parameters, including total hemoglobin concentration and the oxygen saturation (sO_2_) of hemoglobin^20^,^21^. More recently, the functional quantification of oxygen delivery from individual red blood cells (RBCs) was achieved *in vivo* with high temporal-spatial resolution by PAM^22^. To date, OR-PAM has successfully extracted optical absorption-based anatomic, functional, and molecular information, and it demonstrates great potential for studying various physiologies and dynamics in living animals, including vessel anatomy, oxygen metabolism, tumor progression, and brain activities^19^,^20^,^21^,^22^.

Despite the prior success of these microscopic imaging technologies in biomedical research, a single imaging modality alone, such as optical microscopy or PAM, is insufficient to provide a full understanding of the tissue pathophysiology because each can only acquire a certain type of optical imaging information. To overcome this limitation, a multimodal imaging method has been proposed that integrates different imaging modalities into one platform^14^,^20^,^23^. Compared to single-mode imaging, multimodal imaging is capable of extracting various optical properties from the same biological specimens and thus offers complementary anatomic and functional information. Recently developed hybrid microscopic technologies combine PAM with confocal/two-photon microscopy^24^,^25^,^26^, enabling the simultaneous acquisition of both optical absorption and fluorescence imaging information at the cellular level. However, the photoacoustic signal detection configuration was designed in transmission mode for the PAM subsystem^24^,^25^, which restricted its application to only cell samples or thin biological tissue (e.g., the ear or zebrafish embryo). Dong *et al*. integrated PAM into a commercial inverted microscope platform for reflection-mode multimodal microscopic imaging based on an optically transparent micro-ring resonator as an ultrasonic detector^26^; however, *in vivo* imaging was not achieved.

Previously, we configured a novel optical-acoustic imaging probe by combining a high-frequency miniature ultrasonic transducer with a water-immersion objective with a high numerical aperture (NA) of 1.0^19^. Such a design enabled PAM with simultaneous subwavelength transverse resolution and reflection-mode imaging capacity. The volumetric microvascular networks were well resolved *in vivo* in mouse ears. Here, we further developed the *in vivo* multimodal microscopic system, as shown in Fig. 1 (refer to Methods for details about the experimental setup), which combines PAM with two-photon microscopy (TPM) and SHG microscopy to acquire the complementary information of optical absorption, two-photon excited fluorescence, and SHG in small animals.

## Results

### Determination of the spatial resolution of multimodal microscopy

We measured the spatial resolution of the PAM and TPM subsystems, as shown in Fig. 2. Graphite nanoparticles ~80 nm in diameter were imaged by PAM. A representative graphite nanoparticle imaged using PAM is shown in the inset of Fig. 2(a). The photoacoustic amplitude values along the lateral direction of the imaged nanosphere were fitted to a Gaussian function in Fig. 2(a), indicating a PAM lateral resolution of ~290.0 nm according to the full width at half maximum (FWHM). This result agrees with the theoretical diffraction-limited resolution (0.51*λ*/NA ≈271.3 nm) for an NA of 1.0 objective at 532-nm illumination. Although the miniature ultrasonic transducer and the holder bridge were placed under the water-immersion objective, their influences on laser transmission and focusing are essentially negligible because of the optical apodization effect, as demonstrated by simulations in our early work^19^.

To estimate the PAM axial resolution, the photoacoustic signals from diluted India ink solution were recorded using a perpendicular scanning pattern. The peak values of the PA amplitude at each axial position form a one-dimensional depth-resolved profile as shown in Fig. 2(b). Based on the FWHM of Gaussian fitting to the first derivative of the measured raw data, we identified an axial resolution of approximately 3.96 μm. The primary contribution was the highly restricted optical focus of depth (1.8*λ*/NA^2^ ≈1.0 μm) of the high-NA objective.

In Fig. 2(c,d), we characterized the lateral and axial resolutions of TPM, respectively. The TPM image of a fluorescent bead with a diameter of 100 nm is shown in the inset of Fig. 2(c), where we tuned the excitation wavelength to 800 nm. Its fluorescence intensity profile along the lateral direction of the bead was fitted to a Gaussian curve, as shown in Fig. 2(c), indicating a TPM lateral resolution of ~285.7 nm (agreeing well with the theoretical value 0.37*λ*/NA^0.91^ ≈296 nm)^27^. Similarly, the miniature ultrasonic transducer placed under the objective produced a nearly negligible impact on the optical focusing of TPM excitation. The lateral resolution of PAM and TPM is comparable, enabling the transverse co-registration of the pixels acquired by these two imaging modalities. The TPM axial resolution was quantified by perpendicularly scanning a fluorescein solution and recording the fluorescence signals at each axial position. Figure 2(d) shows the measured raw data and the corresponding Gaussian fitting profile obtained by taking its first derivative, which indicated an axial resolution of ~1.13 μm. The theoretical estimation of the axial resolution is 0.63*λ*/[n − (n^2^ − NA^2^)^1/2^] ≈1.11 μm^27^ showing good agreement with the experimental measurement. Due to the nonlinear effect, TPM demonstrated a much finer axial resolution relative to the PAM resolution. Since SHG is a second-order nonlinear optical process, the square-law dependence of SHG signal upon the excitation power will enable a spatial resolution almost the same as that in TPM^28^,^29^. Thus, we can reasonably assume that the SHG resolution is comparable with that of TPM.

### *In vivo* multimodal imaging of mouse ears

The multimodal microscopic images acquired from a nude mouse ear *in vivo* are shown in Fig. 3. In Fig. 3(a), the color-encoded microstructures at different depths were visualized by TPM, SHG, and PAM. By collecting the autofluorescence signals from intracellular NADH, TPM clearly delineated the cell profiles in the left column of Fig. 3(a) [noted by a red solid arrowhead] and Media 1. With the excellent axial resolution of TPM and motorized mechanical depth scanning, these cell clusters in the relatively shallow epidermis layer were identified. Media 1 shows additional epidermis cells at the surface of the mouse ear skin. Additionally, TPM imaged the residual hair shafts [highlighted by red hollow arrowheads in the left column of Fig. 3(a)] due to the intrinsic autofluorescence. Because of the sole noncentrosymmetric feature, the collagen fiber produced strong SHG signals. As a result, SHG microscopy identified the collagen fiber clusters without the need of contrast agents, as shown in the middle column of Fig. 3(a), where a single collagen fiber was distinguished in addition to the spatial interlacing of fibers. The dark regions (noted by the yellow hollow arrowheads) in SHG images originated from the presence of hair follicles (highlighted by the red hollow arrowheads). This finding suggests an automatic registration between these two imaging modalities because they utilize the same illumination light and share the same laser delivery and scanning mechanisms, and the two datasets are acquired simultaneously. Additionally, we observed the collagen fibers underlying the epidermis cells, as shown in Media 1, which corresponded well with the skin anatomy.

Unlike TPM and SHG, which rely on the imaging contrasts of fluorescence and SHG, respectively, optical absorption-based PAM is capable of imaging microvasculature without labels owing to the large optical absorption coefficient of hemoglobin for illumination at 532 nm. In addition to the major blood vessels, capillaries with an estimated diameter less than 10 μm are observed in the right column of Fig. 3(a). PAM characterized the location and distribution of the microvasculature inside the ear, indicating its depth-resolving capability. Most of the vasculature was identified in the dermis layer, as shown in Fig. 3(a) and Media 1. Comparing the images from SHG and PAM at the depth of 64 μm in Fig. 3(a), we found shadows in SHG, along with a high-amplitude PAM image of blood vessels, as highlighted by white arrows, which was primarily caused by severe attenuation of the SHG excitation laser as a result of the high optical absorption of hemoglobin. The co-registration between these images occurred because the illumination lights of PAM and TPM/SHG were well aligned before the *in vivo* imaging and they share the same delivery and scanning mechanism.

In Fig. 3(b), we show the overlaid cross-sectional images from these three imaging modalities, which provide a more comprehensive understanding of the morphology in mouse ears based on the complementary imaging mechanisms. The structural relationships among epidermis cells, hair follicles, collagen fibers, and vascular networks can be visualized clearly in the local environment. Interestingly, we observed hair follicles surrounded by a number of blood vessels in Fig. 3(b) and Media 1. A three-dimensional (3D) microanatomical image was reconstructed by further stacking the cross-sectional images from all depths, as shown in Fig. 3(d), allowing full visualization of the tissue microarchitecture. This technique is typically more advantageous than single-mode imaging technologies. These observations from multimodal microscopy showed good agreement with the skin microanatomy^30^.

### *In vivo* multimodal imaging of the mouse cortex

The brain, one of the most complicated and poorly understood organs, has been avidly studied over the last few decades. Neurovascular coupling is of particular interest in neuroimaging because the constant energy demands during neuronal activity rely on the cerebral vascular system^31^,^32^. Acquisition of both microcirculation and neuron information in the cortex is essential for understanding the underlying mechanism of neurovascular coupling in health or disease.

To comprehensively characterize neurovascular coupling, we conducted *in vivo* multimodal cortex imaging in transgenic thy1-GFP mice, as illustrated in Fig. 4. A series of cross-sectional images of PAM and TPM at varying cortex depths were obtained by scanning the imaging probe to focus the excitation laser at the locations of interest, as shown in Fig. 4(a). Through collection of the intrinsic fluorescence emission from the neurons expressing GFP, TPM was used to visualize the fine neural microstructure, including dendrites and cell bodies, as illustrated in the right column of Fig. 4(a). Nonlinear TPM possesses inherent optical-sectioning imaging capabilities, allowing the depth-resolved visualization of GFP-expressing neurons, as shown in Fig. 4(a). However, fluorescence-based TPM was unable to image the cortex vasculature without contrast agents. The PAM subsystem was used to observe the cortex microvasculature without labels (the left column of Fig. 4(a)) due to the high optical absorption of hemoglobin, which overcame the imposed constraints of administering fluorescent dyes in TPM and enabled the visualization of the brain in its natural state^21^. Therefore, PAM may facilitate the study of cerebrovascular disorders that are commonly associated with cognitive impairments, such as stroke and Alzheimer’s disease^21^,^33^. Note that the depth-resolved information about the anatomic features of the cortex vasculature in PAM was obtained from the restricted optical focal zone (~1 μm in depth) of the high-NA objective. Because of the automatic registration of PAM and TPM, overlying images from these two imaging modalities provide improved insight into the microarchitecture of the cortex vasculature and neurons, as shown in Fig. 4(b), where the vessels and the neurons are pseudo-colored in red and in blue, respectively.

Figure 4(c,d) show the 3D anatomic images of the cortex vasculature and neurons acquired by PAM and TPM, respectively, indicating their volumetric imaging capabilities. To show neural microstructures in greater detail, we presented zoomed-in images [marked with red and yellow dashed squares in Fig. 4(d)] in Fig. 4(f,g), respectively. The neural synaptic structures could be delineated due to the high TPM resolutions. In Fig. 4(f), we clearly identified the dendritic spines (indicated by the red arrows) beneath the cortex surface at 75 μm. The axonal boutons at the depth of 55 μm highlighted by yellow arrows are distinguished in Fig. 4(g). These observations showed good agreement with ref. 32.

In Fig. 4(e), we reconstructed the 3D neurovascular image by merging the raw datasets of PAM and TPM, which provided a comprehensive volumetric visualization of the neurovascular morphology. Media 2 shows the multimodal microscopic 3D neurovascular structure from the different viewing angles. Compared to a single imaging technology alone, such as PAM or TPM, the integration of two technologies enables simultaneous visualization of both the vasculature and neurons. Therefore, this combination is potentially invaluable in the investigation of neurovascular coupling.

## Conclusion

Building on the development of GFP and other fluorescent probes, TPM provides unique molecular information in living organisms at cellular and subcellular resolutions, greatly benefiting research in biology and medicine. Due to its fluorescence-based imaging mechanism, TPM is incapable of imaging nonfluorescent chromophores and substances unless contrast agents are exogenously administered. Relying on the noncentrosymmetric properties of orientated biological components, SHG microscopy delineates the spatial orientation of some microstructures without labels and thus readily complements TPM through the simple introduction of a band-pass filter to record the light signals emitted at half of the excitation wavelength. The limitation of both microscopic technologies, however, is that they neglect optical absorption information, which is specifically associated with the essential physiological and pathological status. In contrast, PAM is based on an optical absorption mechanism and is capable of imaging intrinsic nonfluorescent chromophores, such as hemoglobin, melanin, cytochromes, and lipids, at an extremely high sensitivity within living organisms.

Although three imaging technologies have successfully demonstrated the ability to extract anatomical, functional, molecular, and metabolic information when applied alone, single-mode microscopy techniques, such as TPM, SHG, or PAM, are insufficient to fully evaluate complex tissue because they always fail to detect multiple optical properties of biological samples. To address this problem, we developed a multimodal microscopy technique by integrating PAM, TPM, and SHG into one imaging platform. By detecting complementary imaging information in mouse ears and a transgenic mouse cortex *in vivo*, multimodal microscopy was capable of imaging both fluorescent components (NADH and GFP) and nonfluorescent chromophores (collagen fiber and hemoglobin) down to cellular or even subcellular resolution. With multimodal microscopy, we can obtain a more comprehensive insight into the tissue microarchitecture in living animals, including the vasculature, neurons, and extracellular matrix. A recent study has reported that an amplified femtosecond laser could excite two-photon photoacoustic signal^34^. So in future, we may try to use an amplified femtosecond laser to replace the two independent lasers in this system. With that, the three modality images could be captured simultaneously.

In summary, fully integrated PAM/TPM/SHG multimodal microscopy in reflection mode has been developed and successfully demonstrated to reveal complementary optical properties of the ear and cortex in living mice label-freely. Such an *in vivo* imaging capability may enable a more comprehensive understanding of tissue microarchitecture. In the future, we envision this technology may become a vital tool for unraveling the underlying mechanisms of the complexity, diversity and *in vivo* behaviors of tumor microenvironment and neurovascular coupling.

## Methods

### Experimental setup

**Figure 1:**
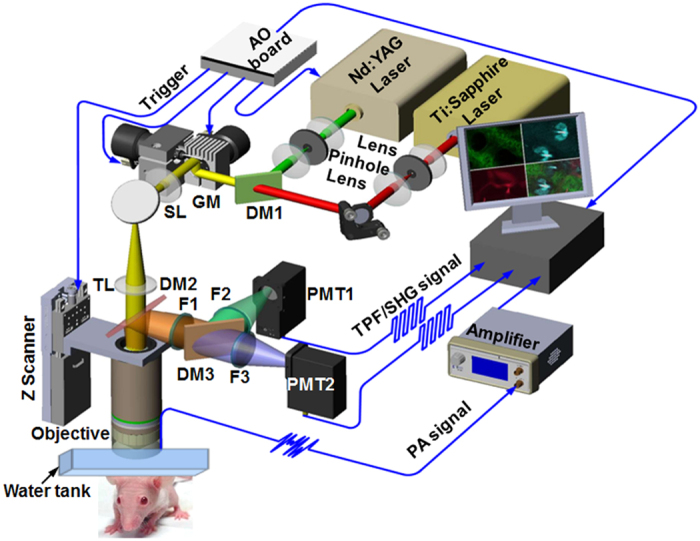
Schematic diagram of the multimodal microscopic system integrating photoacoustic, two-photon, and second harmonic generation microscopies. DM: dichroic mirror; GM: galvanometer; SL: scan lens; TL: tube lens; PMT: photomultiplier.

**Figure 2:**
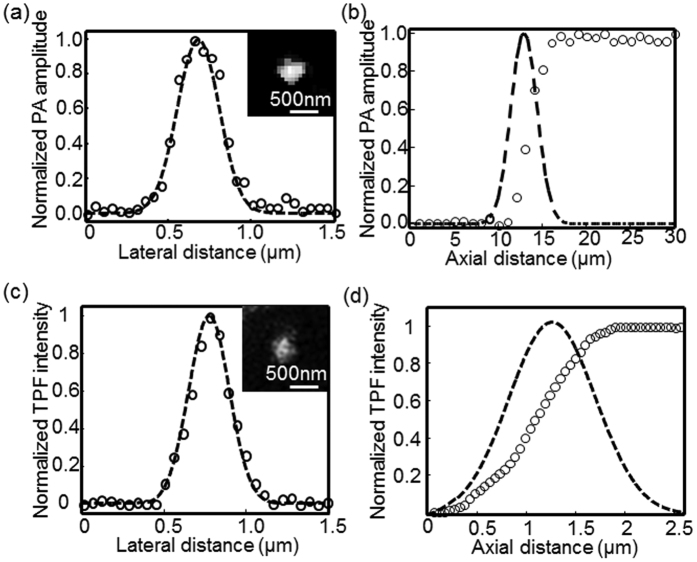
Spatial resolution quantification of multimodal microscopy. (**a**) Lateral resolution of PAM estimated by imaging graphite nanoparticles ~80 nm in diameter. The inset illustrates a representative PAM image of a graphite nanoparticle. (**b**) PAM axial resolution determined by recording PA amplitudes of India ink solution along a one-dimensional depth. Gaussian fitting was performed by taking the first derivative of the measured data. (**c**) The TPM lateral resolution was estimated by imaging fluorescent beads that was 100 nm in diameter. The inset displays a representative nanoparticle acquired by TPM. (**d**) Determination of TPM axial resolution. Fluorescence intensity values of the fluorescent solution along the depth were recorded, and the first derivative was fitted to a Gaussian function. The hollow circles and dashed lines represent the measured raw data and fitted profiles, respectively.

**Figure 3:**
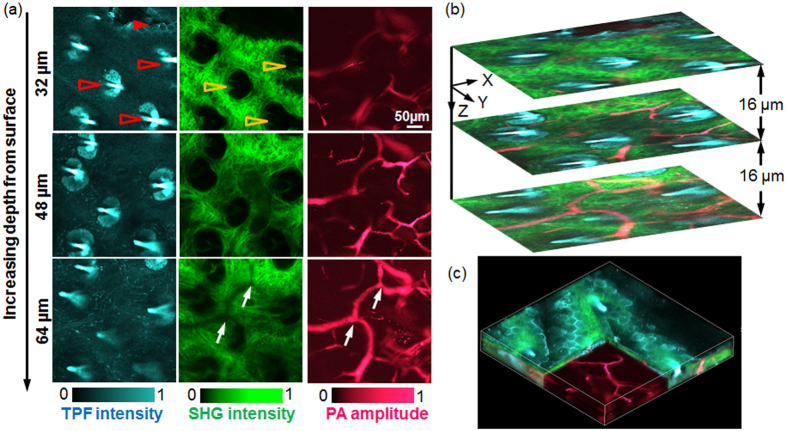
*In vivo* multimodal imaging of a mouse ear. (**a**) Representative depth-resolved color-coded images acquired by TPM, SHGM, and PAM, as indicated. (**b**) Overlaid cross-sectional images of the three imaging modalities. (**c**) Volumetric rendering of the multimodal data sets. Media 1 illustrates the detailed *in vivo* microstructural images from the different depths acquired by multimodal microscopy.

**Figure 4:**
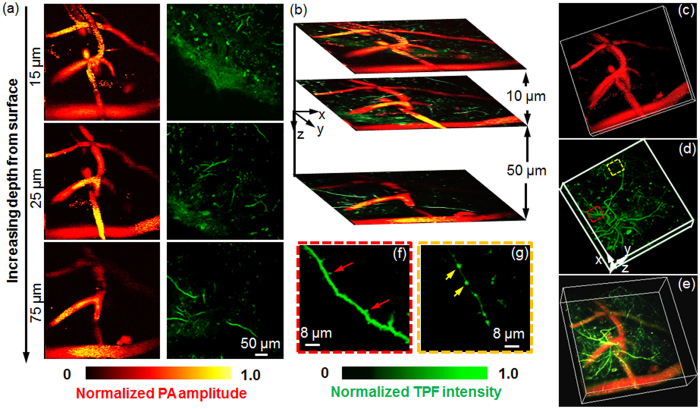
*In vivo* multimodal imaging of the thy1-GFP mouse cortex. (**a**) Color-encoded images of cortex vasculature and neurons at different depths acquired by PAM and TPM. (**b**) Cross-sectional images formed by overlaying the vasculature and neurons from the same depth. (**c**,**d**) 3D visualization of the PAM vascular image and the GFP-expressing neural image from TPM, respectively. (**e**) Overlaid 3D microanatomy reconstructed from PAM and TPM. Media 2 the volumetric rending from different view angles. (**f**,**g**) Close-up images of apical dendrites at depths of 75 μm and 55 μm [highlighted by red and yellow boxes in Fig. 4(d)], respectively.

Figure 1 provides a schematic diagram of the multimodal microscopic system, which combines photoacoustic microscopy (PAM) with two-photon microscopy (TPM) and second harmonic generation (SHG) microscopy into a single imaging platform. A Nd:YAG laser (SPOT-532, Elforlight; wavelength: 532 nm; pulse duration: 1.8 ns) was used as an illumination source for the PAM subsystem; a femtosecond laser (Chameleon Ultra, Coherent) at 800 nm served as the excitation source for the TPM and SHG subsystems. The collimated laser outputs were combined through a dichroic mirror (DM1, Di02-R594, Semrock). A two-dimensional (2D) galvanometer scanner (GM, 6210 H, Cambridge Technology) was used to scan the combined laser beams of PAM and TPM. After passing through a telescope system consisting of a scan lens (SL, f = 50 mm) and a tube lens (TL, f = 250 mm), the laser beams were delivered to the back aperture of a water-immersion objective (XLUMPLFLN 20XW, Olympus; NA = 1.0, working distance: 2 mm). To create an optical diffraction-limited focus spot on the tissue samples, we adjusted the magnification ratio of the telescope system to allow both laser beams to slightly overfill the back aperture of the objective.

For the detection of photoacoustic waves, a customized miniature ultrasonic transducer (central frequency: 42.6 MHz; fractional bandwidth: 60%; length × width × thickness: 0.6 mm × 0.5 mm × 0.2 mm; Blatek, State College, USA) was placed tightly under the center of the optical objective using a homemade steel holder^19^. Because of the optical apodization effect^35^,^36^,^37^, the miniature ultrasonic transducer had a negligible influence on laser focusing and light transmission. As a result, the compact optical-acoustic configuration enabled coaxial laser excitation and ultrasonic detection, as well as high-efficiency collection of fluorescence emission. For more details, refer to our previous paper^19^. During imaging, both the objective and the ultrasonic transducer were immersed in a dish filled with deionized water, and a thin low-density polyethylene film had been applied to the bottom of the dish. This served as an acoustically and optically transparent imaging window. After being amplified by an amplifier (ZFL-500LN-BNC + , Mini-Circuits), the photoacoustic signals were digitized by a data acquisition card at a sampling rate of 250 MS/s (ATS9325, Alazar).

In the TPM and SHG subsystems, another dichroic mirror (DM2, FF510-Di02, Semrock) was inserted between the tube lens and the objective, allowing the transmission of PAM and TPM excitation lasers and the reflection of the emitted fluorescence and SHG signals. After passing through a short-pass filter (F1, FF01-680, Semrock), the emission photons of TPM and SHG were further separated using a dichroic mirror (DM3, Edmund; cut-on wavelength: 435 nm, reflection wavelength: 325–425 nm, transmission wavelength: 444–850 nm). Two photomultipliers (PMT, H7421, Hamamatsu, Japan) were employed to record the two-photon excitation fluorescence and SHG signals. For SHG imaging, a narrow band-pass filter (F3, ET402/15x, Chroma) was placed before PMT2. In the TPM subsystem, we applied a HQ455/70 filter (F2, Chroma) before PMT1 to detect the autofluorescence of NADH, and a band-pass filter (F2, center wavelength: 500 nm, bandwidth: 50 nm, Edmund) was used to collect the GFP fluorescence.

Because the depth of focus was limited owing to the high-NA objective, we mounted the imaging objective to a motorized *z* scanner (MTS25, Thorlabs) for depth scanning. A National Instruments card (PCIe-6320) was used to trigger the laser firing, shutter, and data acquisition of both PAM and TPM/SHG; this board also controlled the scanning of the galvanometer and the *z* scanner.

### Animal preparation

We performed *in vivo* multimodal imaging of mouse ears (nude, 25 g, female) and the thy1-GFP transgenic mouse cortex (25 g, female). To image mouse ears, the animals were first anesthetized by an intraperitoneal (i.p.) injection of 1% W/V sodium pentobarbital solution (150–200 μl). After gentle depilation of the hair of the mouse ear and cleaning of the skin with phosphate-buffered saline, the animals were transferred to a homemade adjustable holder for imaging.

Before cortex imaging, mice were anesthetized with an i.p. injection of sodium pentobarbital, and then the scalp and periosteum were gently removed. A cranial window approximately 2.0 mm in diameter was created to expose the cortex. Immediately after the generation of the cranial window, we immobilized the animal on a homemade imaging holder with a head mount to minimize the motion artifacts caused by breathing. During the imaging experiment, a gas mixture of 1.5% isoflurane and oxygen was continuously ventilated to the mice using a face mask for deep anesthesia. A heating pad was used to maintain the body temperature.

During the multimodal imaging of mouse ears and the cortex, medical ultrasonic gel was applied for the coupling of ultrasound waves; the gel was transparent to the excitation lasers and the emitted fluorescence. All experimental animal procedures were carried out in compliance with the laboratory animal protocols approved by the Animal Studies Committee of the Shenzhen Institutes of Advanced Technology, Chinese Academy of Sciences.

### Image acquisition

To acquire multimodal images, eyepieces (not shown in Fig. 1) were initially used to identify the region of interest in the mouse ears and cortex. Because large excitation power will cause high-order nonlinear photodamage^38^,^39^, we adopted the following imaging acquisition protocol to decrease the instantaneous laser power at the sample. First, we simultaneously acquired the TPM and SHG images while turning off PAM excitation; PAM imaging was activated sequentially while the TPM illumination laser remained off.

In TPM/SHG imaging, we tuned the excitation laser to a wavelength of 800 nm. A high-speed raster scanning pattern consisting of 400 × 400 pixels with a 1-μm interval was implemented in the x-y plane, and this pattern was further combined with motorized depth scanning along the z direction with a 2-μm step. Consequently, a three-dimensional (3D) image was formed. Because TPM and SHG share the same laser source and their signals are detected simultaneously, the total image acquisition time of the TPM/SHG subsystem was primarily determined by the scanning mechanisms. The process required approximately 200 seconds per volume with a line-scanning rate of 100 Hz, and the depth scanning involved 50 positions along the z direction.

Immediately after TPM/SHG imaging, we switched off the illumination light source and relocated the scanning position to the origin. Note that because the high-NA objective had a very limited depth of focus (~1.0 μm), PAM depth information could not be derived from the time of flight of the photoacoustic waves over a large depth range^21^,^22^. To form 3D PAM images with an acceptably large depth, we employed the same scanning pattern as for TPM imaging that combined x-y raster scanning with z axial scanning. Thus, the PAM cross-sectional image was presented with its maximum amplitude projection (MAP) at each depth of interest. The 3D images were reconstructed by stacking all cross-sectional images from different depths together. Due to the limitation of the laser repetition frequency of the photoacoustic excitation source, the line-scanning rate of the PAM subsystem was set at 20 Hz, generating a volumetric dataset within approximately 1000 seconds. Because the illumination lasers of PAM and TPM/SHG were carefully aligned to be coaxial and they shared the same delivery and scanning mechanism, the images from all modalities were laterally co-registered.

## Additional Information

**How to cite this article**: Song, W. *et al*. Fully integrated reflection-mode photoacoustic, two-photon, and second harmonic generation microscopy *in vivo. Sci. Rep.* **6**, 32240; doi: 10.1038/srep32240 (2016).

## Supplementary Material

Supplementary Information

Supplementary Media 2

Supplementary Media 1
