# Supplementary material for: Fully integrated reflection-mode photoacoustic, two-photon, and second harmonic generation microscopy in vivo
Source: Sci Rep. 2016 Aug 31;6:32240. doi: 10.1038/srep32240 (PMC5006040; doi:10.1038/srep32240)
Supplement: Supplementary Information [file srep32240-s1.pdf]

# Fully integrated reflection-mode photoacoustic, two-photon, and second harmonic generation microscopy *in vivo*

Wei Song[1, £], Qiang Xu[1, £], Yang Zhang[1], Yang Zhan[2], Wei Zheng[1, *], and Liang Song[1, *]

[1] *Research Laboratory for Biomedical Optics and Molecular Imaging, Shenzhen Key Laboratory for Molecular Imaging, Institute of Biomedical and Health Engineering, Shenzhen Institutes of Advanced Technology, Chinese Academy of Sciences, Shenzhen 518055, China*

[2] *Brain Cognition and Brain Disease Institute, Shenzhen Institutes of Advanced Technology, Chinese Academy of Sciences, Shenzhen 518055, China*

[£] These authors contributed equally to this work.

* Corresponding authors: zhengwei@siat.ac.cn; liang.song@siat.ac.cn

Media 1. *In vivo* microstructural images of mouse ear from the different depths acquired by multimodal microscopy.

Media 2. *In vivo* volumetric rending of the thy1-GFP mouse cortex from different view angles acquired by multimodal microscopy.
